# Alignment of patient and primary care practice member perspectives of chronic illness care: a cross-sectional analysis

**DOI:** 10.1186/1471-2296-15-57

**Published:** 2014-03-29

**Authors:** Polly H Noël, Michael L Parchman, Ray F Palmer, Raquel L Romero, Luci K Leykum, Holly J Lanham, John E Zeber, Krista W Bowers

**Affiliations:** 1 South Texas Veterans Health Care System, 7400 Merton Minter Blvd, San Antonio, TX 78229, USA; 2 Department of Medicine, University of Texas Health Science Center at San Antonio, 7703 Floyd Curl Dr, San Antonio, TX 78229, USA; 3 MacColl Center for Healthcare Innovation, Group Health Research Institute, Group Health Cooperative, 1730 Minor Ave # 1600, Seattle, WA 98101, USA; 4 The McCombs School of Business, The University of Texas at Austin, 2110 Speedway, Stop B6000, Austin, TX 78712, USA; 5 Central Texas Veterans Health Care System, 1901 S. 1st St, Temple, TX 76504, USA; 6 Scott & White Healthcare Center for Applied Health Research, 2401 S. 31st St, Temple, TX 76508, USA

**Keywords:** Chronic care, Primary care, Patient surveys

## Abstract

**Background:**

Little is known as to whether primary care teams’ perceptions of how well they have implemented the Chronic Care Model (CCM) corresponds with their patients’ own experience of chronic illness care. We examined the extent to which practice members’ perceptions of how well they organized to deliver care consistent with the CCM were associated with their patients’ perceptions of the chronic illness care they have received.

**Methods:**

Analysis of baseline measures from a cluster randomized controlled trial testing a practice facilitation intervention to implement the CCM in small, community-based primary care practices. All practice “members” (i.e., physician providers, non-physician providers, and staff) completed the Assessment of Chronic Illness Care (ACIC) survey and adult patients with 1 or more chronic illnesses completed the Patient Assessment of Chronic Illness Care (PACIC) questionnaire.

**Results:**

Two sets of hierarchical linear regression models accounting for nesting of practice members (N = 283) and patients (N = 1,769) within 39 practices assessed the association between practice member perspectives of CCM implementation (ACIC scores) and patients’ perspectives of CCM (PACIC). ACIC summary score was not significantly associated with PACIC summary score or most of PACIC subscale scores, but four of the ACIC subscales [Self-management Support (p < 0.05); Community Linkages (p < 0.02), Delivery System Design (p < 0.02), and Organizational Support (p < 0.02)] were consistently associated with PACIC summary score and the majority of PACIC subscale scores after controlling for patient characteristics. The magnitude of the coefficients, however, indicates that the level of association is weak.

**Conclusions:**

The ACIC and PACIC scales appear to provide complementary and relatively unique assessments of how well clinical services are aligned with the CCM. Our findings underscore the importance of assessing both patient and practice member perspectives when evaluating quality of chronic illness care.

**Trial registration:**

NCT00482768

## Background

The Chronic Care Model (CCM) has become a widely accepted framework for organizing and delivering patient-centered, evidence-based care for patients with chronic illnesses within the primary care setting [1]. The model describes six elements that health care organizations need to optimize chronic illness care: decision support, self-management support, clinical information systems, delivery system design, organizational leadership and support, and community linkages [2]. A basic premise of the CCM is that health care settings where these elements are robust are likely to have prepared, proactive practice teams, and informed, engaged patients who become active members of their health care teams and accept shared responsibility for their chronic illness care [2]. As a result of productive interactions between these teams and patients, both the quality and outcomes of care for patients with chronic illness should improve.

Assessments of the degree to which care is consistent with the CCM originally focused on provider and system perspectives using tools such as the Assessment of Chronic Illness Care (ACIC) survey [3]. Studies indicate that in practices with higher ACIC scores, patients with diabetes have better glucose control and lower risk of cardiovascular complications [4,5]. Limited data also suggest that the ACIC is responsive to quality improvement changes for diabetes [6,7]. Glasgow and colleagues later developed the Patient Assessment of Chronic Illness Care (PACIC) to measure aspects of chronic illness care from the patient perspective [8,9]. For chronic illness care delivery, the degree to which elements of the CCM have been implemented should influence the care experience of the patient with a chronic illness. However, to date, little is known as to whether primary care teams’ perceptions of how well they deliver care consistent with the CCM corresponds with their patients’ own experience of chronic illness care.

There is growing interest in measuring and evaluating patient experiences of their care as an important dimension of quality [10]. Going beyond traditional measures of “satisfaction”, tools such as the PACIC assess specific experiences of patients with the care they receive relevant to their underlying conditions and the setting where they receive their care. There is emerging evidence that such patient-reported measures are strongly correlated with better outcomes [11-13]. The delivery of care for a chronic illness is after all a service, and as such the patient’s experience of the service rendered should be included in any overall assessment of quality.

We examined the extent to which practice members’ perceptions of how well they have organized to deliver care consistent with the CCM were associated with their patients’ perceptions of chronic illness care in small, community-based primary care clinics. We hypothesized that the overall degree to which primary care providers and staff report that their delivery of chronic illness care is consistent with the CCM would be associated with their patients’ experience of chronic illness care, controlling for patient characteristics and nesting of primary care practice members and patients within primary care practices. In addition, we were also interested in determining whether specific elements or components of care consistent with the CCM were associated with patient-reported experience of care.

## Methods

### Study design and setting

We used baseline surveys from a cluster randomized controlled trial testing a practice facilitation intervention to implement the CCM. The study design of this trial and details about the intervention have been previously reported [14]. Briefly, the study was conducted at 39 small, primary care clinics or “practices” in South Texas. These small urban, suburban and rural practices, each with one to three physicians, serve a population of patients diverse in demographic characteristics, insurance coverage, and health care needs. The intervention consisted of an external practice facilitator who met at least monthly for up to one year with all of the members of each practice to assist them with CCM implementation [14].

### Partcipants and data collection

Subjects for this analysis included the primary care practice members (i.e., the physician providers, non-physician providers, and staff) at participating primary care practices and their patients. After study enrollment but prior to initiation of the facilitation intervention, we conducted baseline evaluations at each practice. These included a “Practice Member Survey” that was distributed to all the providers and staff at each practice. The Practice Member Survey included the ACIC [3], as well as individual items assessing the sociodemographic and professional characteristics of the practice members. We also collected information about practice characteristics and operations. All practice member participants provided informed consent for the study, which was approved by the Institutional Review Board at the University of Texas Health Science Center at San Antonio.

In addition, we asked convenience samples of adult visitors at each participating practice to complete patient surveys as they checked-in. The patient survey included the PACIC [8], as well as other measures described below. Because the majority of clinics served communities with substantial Hispanic populations, both English-language and Spanish-language versions of the survey were made available to patient participants. We asked each person to indicate the purpose of their clinic visit; for this analysis we excluded those who reported they were only accompanying another person (e.g., child or elderly parent) who had an appointment. Of the patients who remained, we included those who reported that they had 1 or more chronic illnesses. The patient surveys, which were anonymous, were accompanied by an IRB-approved “Information Sheet” which described the study and participants’ rights as research subjects.

### Practice member measures

#### ACIC survey

The extent to which the care delivered in each practice is consistent with the elements of the CCM was measured with the ACIC [3]. The ACIC is a 25-item survey that measures the presence of the 6 elements of the CCM. Each item is scored on a 0 to 11 scale and provides sub-scale scores for each of the 6 CCM components as well as a summary score. Scores from 0 to 2 represent “limited or no support for chronic illness care”, 3 to 5 represent “basic or intermediate support”, 6 to 8 is “advanced support”, and 9 to 11 represent “optimal or comprehensive, integrated care for chronic illness” [3]. Version 3.5 of the ACIC was used in this study [15]; in addition to the six subscales, it also includes items that address how well a practice integrates the CCM elements into daily patient care.

#### Professional role of practice members

In addition to the measures above, the Practice Member Survey also instructed practice members to indicate which professional role they served in the practice from the following list: Physician, Physician Assistant (PA), Nurse Practitioner (NP), RN, LVN, Medical Assistant, Receptionist, Office Manager, and “Other”.

#### Practice environment checklist

A Practice Environment Checklist (PEC) was completed by the lead physician or office manager to capture descriptive information about the characteristics and operations of each practice in a structured format. The PEC was adapted from similar checklists utilized in studies of preventive service delivery in primary care practices [16-18]. Information collected included the number of practice members, number of office visits per day per full-time employee, and whether or not medical records are computerized, as well as characteristics of the practice’s patient population: percent of minorities, and percent of patients using Medicare or Medicaid.

### Patient measures

Sociodemographic Characteristics and Health-related Measures: The patient survey included items to assess sociodemographic characteristics, including sex, race/ethnicity, and education. The first item from the Medical Outcomes Study Short-Form (SF) 36 [19], commonly referred to as the SF1 and generally accepted as a valid measure of general health status, was used to assess self-reported health [20]. Chronic illnesses were also self-reported by patients in the first nine clinics in response to the question: *“Has a doctor or other health professional had ever told you that you have diabetes (yes/no) or other chronic diseases such as high blood pressures, high cholesterol, depression, asthma, emphysema, etc. (yes/no)”.* In the remaining clinics, this question was subsequently refined to include a checklist of diabetes and 19 other common chronic illnesses.

#### PACIC

The PACIC was developed to measure the extent to which patients with chronic illness receive clinical care that is patient-centered, proactive, and planned, and that includes collaborative goal-setting, problem-solving, and follow-up support [8]. The 20-item survey consists of 5 subscales representing components of the CCM as experienced by patients: 1) Patient Activation; 2) Delivery System Design/Decision Support; 3) Goal-setting; 4) Collaborative Problem-solving; and 5) Follow-up & Coordination. Patients rate each item with a 5-point scale indicating how often they experience the clinical care or service described. Ratings are averaged to yield subscale scores and a summary score; scores range from 1-5 with higher scores indicating patients’ perception that their care is more consistent with the CCM.

### Analytic plan

Descriptive statistics were assessed on practice member and patient characteristics, and ACIC and PACIC scales. Subscale scores for respondents with missing values were included only if they responded to a majority of items that comprised the subscale. Multiple imputation was used for missing scale items, which ranged from 0% to 6% for ACIC subscales and 0% to 5% for PACIC subscales in our data. Multiple imputation is a widely recommended strategy replacing missing data, and has been found to produce less bias in regression coefficients and standard errors than mean substitution [21,22]. Items were averaged for subscale scores and summary scores for both the PACIC and ACIC were then created by summing and averaging their respective subscales. We calculated Cronbach’s alpha for ACIC and PACIC summary scores and their subscale scores to assess internal consistency.

To assess the association between practice member perspectives of CCM implementation (ACIC scores) and patients’ perspectives of CCM (PACIC), we used hierarchical linear regression models to account for nesting of practice members and patients within practices. This was done to obtain unbiased standard errors of the regression coefficients. Intraclass correlation coefficients (ICCs) were derived from the variance estimates of hierarchical models by dividing the level 2 variance by the total variance. This yielded estimates of the degree to which individuals in the same clinics responded similarly to the ACIC and PACIC. ICCs greater than .05 indicate that the data should be analyzed using hierarchical methods in order to avoid artificially low standard errors of the regression estimates [23].

Two sets of regression models were created. In the first set, the ACIC summary score and each ACIC subscale score were regressed separately onto the PACIC summary score and each subscale score. In the second set of models, patients’ sociodemographic characteristics (i.e., gender, age, race/ethnicity, and education) and self-reported health status were added as covariates to each hierarchical regression model. In a prior multivariate analysis of our data, the only practice characteristic that was significantly associated with ACIC scores was the presence of an electronic medical record (EMR) [24]. We therefore conducted sensitivity analyses stratified by practices that did and did not have EMRs. MlWin software was used for the hierarchical regression analysis [25].

## Results

A total of 40 practices were recruited for participation in the study. Early recruits included 10 active members of a primary care Practice-Based Research Network (PBRN), all of whom agreed to participate. PBRN enrollees were asked to recommend colleagues whom they thought might be interested in study participation who also referred colleagues (n = 25). These physicians were contacted directly by phone and in-person recruiting visits were scheduled at their offices resulting in 22 participants. In addition, 145 recruitment letters were sent to primary care physicians within the region identified from professional society membership guides. From these letters 15 practices responded, of those eight agreed to participate in the study, resulting in 40 practices that were enrolled and randomized in the study. One small practice, however, withdrew from the study due to a re-organization before baseline measures were completed.

Of the 39 remaining practices, 32 were led by a single physician, six were led by two physicians, and one practice had three physicians. Additional practice characteristics are reported in Table 1. A total of 291 of 296 practice members (98%) completed baseline surveys. Eight of the 291 practice members were later determined to be temporary or part-time employees (e.g., contract workers or students), and the surveys of these cases were excluded. The professional categories (e.g., physician, non-physician providers, direct care staff, and non-direct care staff) of the 283 practice members are reported in Table 1.

**Table 1:** Characteristics of participating patients, practice members, and primary care practices

**Primary care patients with 1 or more chronic illnesses (N = 1,886)**	
Age in years, mean (SD)	55.1 (15.2)
Female (%)	65.0
Ethnicity	
White non-Hispanic (%)	39.7
Hispanic (%)	48.7
Black (%)	5.5
Other (%)	6.1
Education	
High school graduate/GED or less (%)	42.6
High school graduate with some college (%)	31.0
College graduate (%)	26.4
Self-rated health, mean (SD)	3.0 (0.9)
**Practice Members (N = 283)**	
Physicians (%)	15.8
Non-physician primary care providers (PAs, NPs)	6.5
Direct care staff (%)	43.7
Non-direct care staff (%)	34.1
**Practices (N = 39)**	
Number of providers, M (SD)	2.17 (1.1)
Number of non-provider staff, M (SD)	8.5 (6.8)
Office Visits per Day per FTE, Mean (SD)	23.0 (5.5)
Percentage of Medicaid Patients, M (SD)	12.3 (16.2)
Percentage of Medicare Patients, M (SD)	32.8 (21.5)
Percentage of White Patients, M (SD)	28.9 (18.7)
Percentage of Practices with EMR (%)	51.3

A total of 2,634 individuals from the 39 clinics were invited to complete patient surveys. Of the 2,493 individuals who returned usable surveys (94.6% response rate), 607 were not eligible because they were accompanying another person for an appointment and/or because they did not report having a chronic illness, leaving a sample of 1,886 patients [range 17 to 81 per practice site; 48.4 mean (13.5 SD)] meeting eligibility criteria. The majority of these individuals were female, of Hispanic or other racial/ethnic background, and had not graduated from college (see Table 1).

### PACIC and ACIC scores

The mean PACIC summary and subscale scores for the patient participants and the ACIC summary and subscale scores for the practice members are reported in Table 2. The mean PACIC summary score was 3.22 (SD 1.12), indicating that on average, patients experienced clinical care that was consistent with the CCM “some of the time”. Among the PACIC subscales, average scores were highest for the Decision Support subscale (M = 3.69; SD = 1.1) and lowest for the Follow-up/Coordination subscale (M = 2.69; SD = 1.3). The mean ACIC summary score was 6.2 (SD 2.1), indicating that on average, the practice members rated their chronic illness care delivery in the “Level B” or “advanced support” range. Among the ACIC subscales, average scores were highest for the Organizational Support subscale [Mean (SD) = 6.8(2.4)] and lowest for the Clinical Information Systems subscale [Mean (SD) = 5.8(2.6)].

**Table 2:** Mean (SD), alpha coefficients, and ICC for PACIC & ACIC summary and subscale scores

**PACIC Scores (N = 1,866) Range of possible scores 0 to 5**			
**Scale/Subscale**	**Mean (SD)**	**α**	**ICC**
Summary	3.22 (1.1)	0.95	.08
Patient Activation/Involvement	3.39 (1.3)	0.85	.07
Delivery System Design/Decision support	3.69 (1.1)	0.77	.04
Goal-setting/Tailoring	3.13 (1.3)	0.86	.05
Problem-solving/Contextual	3.53 (1.3)	0.89	.05
Follow-up/Coordination	2.69 (1.3)	0.88	.08
**ACIC Scores (N = 286) Range of possible Scores 0 to 11**			
**Scale/Subscale**	**Mean (SD)**	**α**	**ICC**
Summary	6.2 (2.1)	0.94	.11
Decision Support	6.0 (2.5)	0.80	.18
Self-management Support	6.2 (2.4)	0.84	.06
Community Linkages	6.1 (2.6)	0.74	.10
Clinical Information Systems	5.8 (2.6)	0.86	.11
Delivery System Design	6.5 (2.2)	0.85	.16
Organizational Leadership & Support	6.8 (2.4)	0.88	.16
CCM Integration	6.0 (2.5)	0.89	.10

The two measures of chronic illness care demonstrated good internal consistency, with alpha coefficients for the ACIC subscales ranging from .74 to .89, and from .77 to .89 for PACIC subscales. The ICC scores for the PACIC and ACIC summary scores and all subscales for both measures were greater than .05, indicating that they should be analyzed using hierarchical methods to control for clustering of observations within practices and avoid artificially low standard errors of the regression estimates [23].

### Predictors of PACIC

Table 3 provides the unadjusted and adjusted regression coefficients for each model with the ACIC summary score and ACIC subscale scores regressed onto the PACIC summary scores and PACIC subscale scores. The only patient-level covariate that was significantly associated with PACIC summary score and all PACIC subscale scores was education, such that patients with lower levels of educational attainment were more likely to report better chronic illness care. In addition, specific racial/ethnic subgroups were also significantly associated with PACIC scores, but these varied across the summary and subscale scores.

**Table 3:** Unadjusted and adjusted regression coefficients with standard errors from hierarchical regression models for ACIC predicting PACIC in patients with one or more chronic diseases (n = 1769).

	**PACIC (Dependent variable)**					
**ACIC Predictor**	**Summary**	**Patient activation**	**Delivery system design/Decision support**	**Goal-setting**	**Problem-solving**	**Follow-up/Coordination**
*Summary*	.059 (.034)^ns^	.057 (.039)^ns^	.011 (.031)^ns^	.064 (.037)^ns^	.056 (.038)^ns^	.084 (.039)^*^
	**.050 (.033)**^**ns**^	**.066 (.036)**^**ns**^	**.011 (.029)**^**ns**^	**.055 (.035)**^**ns**^	**.056 (.036)**^**ns**^	.076 (.036)^*^
*Decision support*	.026 (.027)^ns^	.035 (.031)^ns^	-.010 (.024)^ns^	.027 (.029)^ns^	.026 (.030)^ns^	.042 (.031)^ns^
	**.030 (.025)**^**ns**^	**.044 (.028)**^**ns**^	**-.007 (.023)**^**ns**^	**.028 (.027)**^**ns**^	**.030 (.029)**^**ns**^	**.045 (.029)**^**ns**^
*Self-management support*	.084 (.033)^*^	.091 (.037)^*^	.048 (.030)^ns^	.099 (.035)^*^	.078 (.037)^*^	.084 (.038)^*^
	.088 (.032)^*^	.099 (.034)^*^	**.052 (.028)**^**ns**^	.099 (.033)^*^	.081 (.035)^*^	.088 (.035)^*^
*Community linkages*	-.082 (.024)^**^	-.100 (.028)^**^	-.072 (.022)^*^	-.088 (.026)^**^	-.093 (.027)^**^	-.065 (.029)^*^
	-.077 (.023)^**^	-.080 (.027)^**^	-.069 (.022)^*^	-.086 (.025)^**^	-.087 (.026)^**^	-.066 (.028)^*^
*Clinical information systems*	.069 (.029)^*^	.079 (.033)^*^	.012 (.026)^ns^	.066 (.031)^*^	.068 (.033)^*^	.097 (.033)^**^
	.060 (.027)^*^	.073 (.030)^*^	**.009 (.025)**^**ns**^	**.054 (.030)**^**ns**^	**.062 (.032)**^**ns**^	.086 (.031)^**^
*Delivery system design*	.108 (.030)^**^	.117 (.034)^**^	.053 (.027)^ns^	.112 (.032)^***^	.107 (.034)^*^	.132 (.034)^**^
	.094 (.028)^**^	.108 (.031)^**^	**.044 (.026)**^**ns**^	.094 (.031)^**^	.096 (.032)^*^	.114 (.032)^**^
*Organizational support*	.101 (.030)^***^	.088 (.035)^*^	.059 (.028)^*^	.119 (.033)^**^	.104 (.035)^*^	.117 (.035)^**^
	.097 (.028)^**^	.098 (.032)^*^	**.056 (.027)**^**ns**^	.106 (.031)^*^	.102 (.033)^*^	.097 (.034)^**^
*CCM Integration*	.059 (.028)^ns^	.047 (.033)^ns^	.017 (.026)^ns^	.063 (.031)^*^	.062 (.032)^*^	.083 (.033)^*^
	**.053 (.027)**^**ns**^	**.054 (.030)**^**ns**^	**.015 (.025)**^**ns**^	**.051 (.029)**^**ns**^	**.060 (.031)**^**ns**^	.071 (.031)^*^

***p < .001; **p < .02; *p < .05); + = p < .10.

Regression coefficients in the first row of each cell are unadjusted; those on the second row in each cell in **BOLD** are adjusted for age, gender, education, race, & self-reported health.

After controlling for patient-level characteristics, ACIC summary scores were not associated with PACIC summary scores or any of the PACIC subscale scores except Follow-up/Coordination. Of the ACIC subscale scores, Self-management Support, Community Linkages, Delivery System Design, and Organizational Support were significantly associated with PACIC summary scores and the majority of PACIC subscale scores after controlling for patient-level characteristics, but the magnitude of the association was relatively weak (Table 3). For example, a 1-point increase in mean Delivery System Redesign subscale score on the ACIC was only associated with a 0.11 increase with the mean PACIC summary score. Interestingly, the association between the Community Linkages subscale and the PACIC summary score and subscales was inverse. Of the remaining ACIC subscales, Clinical Information Systems was significantly associated with the PACIC Summary score, but only two of the five PACIC subscales, while the CCM Integration subscale of the ACIC was significantly associated with the PACIC Follow-Up/Coordination subscale only. The ACIC Decision Support subscale was not significantly associated with any adjusted PACIC subscale or summary scores. None of the ACIC subscales were significantly associated with adjusted scores from the Delivery System Design/Decision Support subscale of the PACIC. Sensitivity analyses indicated that there were no differences in regression coefficients for practices that had EMRs and those that did not.

## Discussion

Although the PACIC was designed to complement the ACIC by providing a tool for assessing patients’ perspectives of their chronic illness care experience, there have been no previously published peer-reviewed reports of how well patient and practice members’ perceptions of chronic illness care are aligned. Our findings indicate that the ACIC summary score was not associated with PACIC summary scores or the majority of PACIC subscale scores. These findings indicate that practice members’ overall assessment of the degree to which the CCM has been implemented, as measured by the ACIC, is not meaningfully associated with patients’ evaluation of their own care experiences. The findings also suggest that patients’ global perceptions of their chronic illness care, as assessed by the PACIC, and more specifically their receipt of care promoting patient activation, goal-setting, problem-solving, and care coordination, are associated with key elements of chronic illness care, such as self-management support, delivery system design, and organizational support provided in the primary care clinics where they receive their care, as assessed by the clinicians and other staff in these practices. The level of association, however, is weak, underscoring the importance of assessing both patient and practice member perspectives when evaluating quality of chronic illness care.

The ACIC elements that were most consistently associated with patient experiences appear to reflect more inter-relational aspects of good chronic illness care such as the Self-management Support element, team-based care suggested in the Delivery System Design element, and leadership support apparent in the Organizational Support element of the CCM. In contrast, the CCM elements of Decision Support for clinicians, such the availability of evidence-based guidelines and integration of specialists; Clinical Information Systems indicating the use of tools such as disease registries, reminders, and feedback to improve care; and the overall CCM Integration into clinic operations were less consistently associated with patients’ experiences of their chronic illness care.

As has been previously noted, the 5 PACIC subscales do not map perfectly onto the 6 CCM components as measured by the ACIC [8]. According to the developers of the PACIC instrument, most patients may not be aware of some aspects of their chronic illness care, such as clinical information systems or organization of health care [8]. In our study, however, these two subscales of the ACIC were significantly associated the PACIC summary score and some of the PACIC subscales. In contrast, the Delivery System Design/Decision Support subscale of the PACIC was not associated with either the Delivery System Design subscale or the Decision Support subscale of the ACIC, or the summary score or any other subscale score of the ACIC.

It is also noteworthy that assessments of linkages to community resources by clinicians and staff were inversely associated with patients’ reports of their chronic care experiences. In reviewing our qualitative notes for the study, it appears that clinics with high scores on the Community Linkages subscale of the ACIC tended to serve more rural and lower socioeconomic status populations. These clinics tend to be under-resourced and to have higher patient volumes. Although speculative, it is possible that it was difficult for these clinics to effectively coordinate and incorporate these community resources into their patients’ chronic illness care, especially if patients had difficulty finding transportation or lacked insurance. Due to increasing interest in linking primary care with community resources [26,27], this finding deserves further exploration in future research.

Although the validity of ACIC has previously been supported in several studies, these have varied methodologically with regard to the respondents who completed the measure. For example, some of the studies have restricted respondents to clinicians (e.g., physician, nurse practitioners, and physician assistants) [4,28]. Other studies employing the ACIC have utilized ratings by general practitioners, non-physicians, and policy and management professionals [29], teams consisting of clinic physicians, nurse manager/coordinators, and administrative decision makers [3], physicians and staff [5], clinics’ administrative supervisors [30], and unspecified site participants [31]. Some may question non-clinicians’ ability to accurately judge the quality of chronic illness care; however, the present findings suggest that several subscales of the ACIC as rated by diverse teams of practice members ranging from physicians to front office staff are weakly associated with patients’ own perceptions of their chronic illness care. These results build upon prior literature suggesting that the ACIC is a valid measure of chronic illness care that is associated with a broad array of patient, provider, and organizational outcomes [3-5,24,28-32].

A 2010 review of the PACIC found conflicting results about its purported 5-factor structure and suggested that a single factor interpretation was more appropriate, raising questions about the utility of its subscales [33]. The authors suggested that the PACIC is a formative rather than a reflective measure, which renders such assessments inappropriate, and recommended that future evaluations focus on its construct and criterion validity [33]. Although the present findings provide only minimal support for the construct validity of the PACIC in relation to the ACIC, several recent randomized controlled trials indicate that patients who received health care services that had organized to provide better chronic illness care (i.e., disease management program for patients with COPD, “guided” care for older patients multimorbidity, and group visits for patients with diabetes), reported higher PACIC scores at study, compared to patients who received usual care services [34-36]. Additional research is needed to determine the extent to which the PACIC is related to patient outcomes; nevertheless, patient experience is increasingly recognized as an important outcome in and of itself [10].

Prior research has also been mixed as to whether or not patient demographic characteristics are associated with PACIC scores [8,37,38]. When differences have occurred, as in our findings where patients with lower levels of education compared to those with higher levels, and African Americans and other minorities, compared to non-Hispanic whites, were more likely to report that their medical care was consistent with the CCM, it is not clear whether this reflects actual differences in chronic illness care delivery or differences in the perception of care [38].

Our findings are limited by the cross-sectional nature of the analysis, restriction to a narrow geographical region of the United States, and selection bias inherent in the convenience samples of patients utilized for the study. In addition, the participating primary care practices were small, and we must acknowledge that different results might have been obtained using larger primary care clinics, especially those embedded within integrated health care systems. Furthermore, although the analyses were adjusted for patient demographics and self-reported health status, we were not able to control for number of chronic illnesses or other factors that might be associated with perceptions of chronic illness care, such as patient utilization. It is also possible that different results may have been obtained among a more homogeneous patient sample having a specific chronic illness, such as diabetes. In spite of these limitations, however, the study has a number of strengths, including a large, diverse patient sample drawn from multiple clinics and the use of hierarchical modeling.

## Conclusions

Improving patient experiences of care is a priority within the National Quality Strategy as reflected in recent multi-payer initiatives that include the use of patient experience results in determining provider payment [39]. This analysis provides the first published report exploring the association between how chronic illness care is organized and delivered from the perspective of those providing the care, and patient-reported care experiences. The ACIC and PACIC scales appear to provide complementary, but relatively unique assessments of how well clinical services are aligned with the CCM. Our findings underscore the importance of assessing both patient and practice member perspectives when evaluating quality of chronic illness care.

## Abbreviations

CCM: Chronic care model; ACIC: Assessment of chronic illness care; PACIC: Patient assessment of chronic illness care; RN: Registered Nurse; LVN: Licensed vocational nurse; NP: Nurse Practitioner; PA: Physician assistant; ICC: Intraclass correlation coefficient.

## Competing interests

The authors declare that they have no competing interests.

## Authors’ contributions

MLP designed the clinical trial; MLP and RLR led day-to-day study management. PHN conceptualized this analysis of baseline data. PHN, MLP, RLR, RFP, LKL, HJL, JEZ, and KWB provided input into data interpretation. RFP performed the analyses. All authors contributed to manuscript writing and read and approved the final manuscript.

## Pre-publication history

The pre-publication history for this paper can be accessed here:

http://www.biomedcentral.com/1471-2296/15/57/prepub
